# The imperative for multigenerational genetic screening: A case report of fragile X-associated tremor/ataxia syndrome (FXTAS)

**DOI:** 10.1097/MD.0000000000045411

**Published:** 2025-10-17

**Authors:** Yueqi Huang, Ziyang Huang, Zheng Wang, Wenxin Tang, Xiaoying Zhang

**Affiliations:** a Affiliated Mental Health Center & Hangzhou Seventh People’s Hospital, Zhejiang University School of Medicine, Hangzhou, Zhejiang Province, China.

**Keywords:** cognitive impairment, fragile X-associated tremor/ataxia syndrome (FXTAS), fragile X-syndrome (FXS), multigenerational genetic screening, neuronal intranuclear inclusion disease (NIID)

## Abstract

**Rationale:**

Fragile X-associated tremor/ataxia syndrome (FXTAS), presenting with cognitive impairment as the initial symptom, is rare. This report emphasizes the need to consider FXTAS diagnosis in cases of early onset cognitive impairment in an aging population.

**Patient concerns:**

A 57-year-old male with FXTAS was initially misdiagnosed with neuronal intranuclear inclusion disease, whose first manifestation was cognitive impairment. Testing showed a verbal IQ of 74, a performance IQ of 73, and a full scale of IQ 71, and a Clinical Memory Quotient of 74. Furthermore, his Mini-Mental State Examination score of 23 reflected a decline in short-term memory. Following reevaluation of imaging, identified T2-fluid attenuation inversion recovery hyperintensity at the cerebellar peduncles, and further investigation of the family history revealing a 7-year-old grandson with fragile X syndrome (FXS), repeat genetic testing of the patient demonstrated 121 CGG repeats in the FMR1 gene, confirming the diagnosis of FXTAS.

**Diagnoses:**

FXTAS.

**Interventions:**

The patient was treated with donepezil and simvastatin daily and alcohol consumption was restricted.

**Outcomes:**

After 1 year, the patient showed partial improvements in memory, with his Mini-Mental State Examination score rising to 27, allowing him to resume employment as a community security guard.

**Lessons:**

Due to the highly variable clinical presentation of FXS within families, clinicians should always consider fragile X testing and detailed family history when middle-aged and elderly males exhibit unexplained cognitive impairment or tremors. With the acceleration of aging in society, this case underscores the importance of multigenerational genetic screening for maternal grandparents, particularly males, in FXS families and prioritizing follow-up monitoring.

## 1. Introduction

Fragile X-associated tremor/ataxia syndrome (FXTAS) is caused by FMR1 premutation,^[1]^ typically presenting initially with tremor, while ataxia or cognitive impairment generally manifests later.^[2,3]^ The disorder exhibits significant clinical heterogeneity, posing diagnostic challenges when cognitive impairment is the initial presentation in middle-aged and older males.

In recent years, studies have found that the risk of premutation-to-full mutation (FM) expansion is correlated with maternal transmission. Female premutation carriers have up to a 50% risk of transmitting FM to their offspring, prompting healthcare institutions to strengthen cross-generational genetic counseling for offspring.^[4]^ Research has shown that when a child is diagnosed with fragile X syndrome (FXS) and the mother carries a premutation allele, the maternal grandfather is at a heightened risk of developing FXTAS.^[5]^ However, clinical practice has yet to emphasize the necessity of cross-generational grandparental screening. This case report describes a tortuous diagnostic process of a middle-aged male initially misdiagnosed with neuronal intranuclear inclusion disease (NIID), whose FXTAS diagnosis was ultimately confirmed through meticulous family history tracing, underscoring the significance of emphasizing family history and cross-generational genetic screening.

## 2. Case presentation

A 57-year-old male presented with progressive short-term memory decline for over 1 year. Symptoms progressively included mild dizziness and occasional bilateral lower limb static tremors during stair-climbing. Past medical history included a prior diagnosis of “hyperlipidemia,” which was managed with simvastatin 20 mg/d. personal history revealed a 20-pack-year smoking habit (5–6 cigarettes/d) and occasional alcohol use. The patient denied a family history of neurological disease at the initial visit.

**Figure 1.**
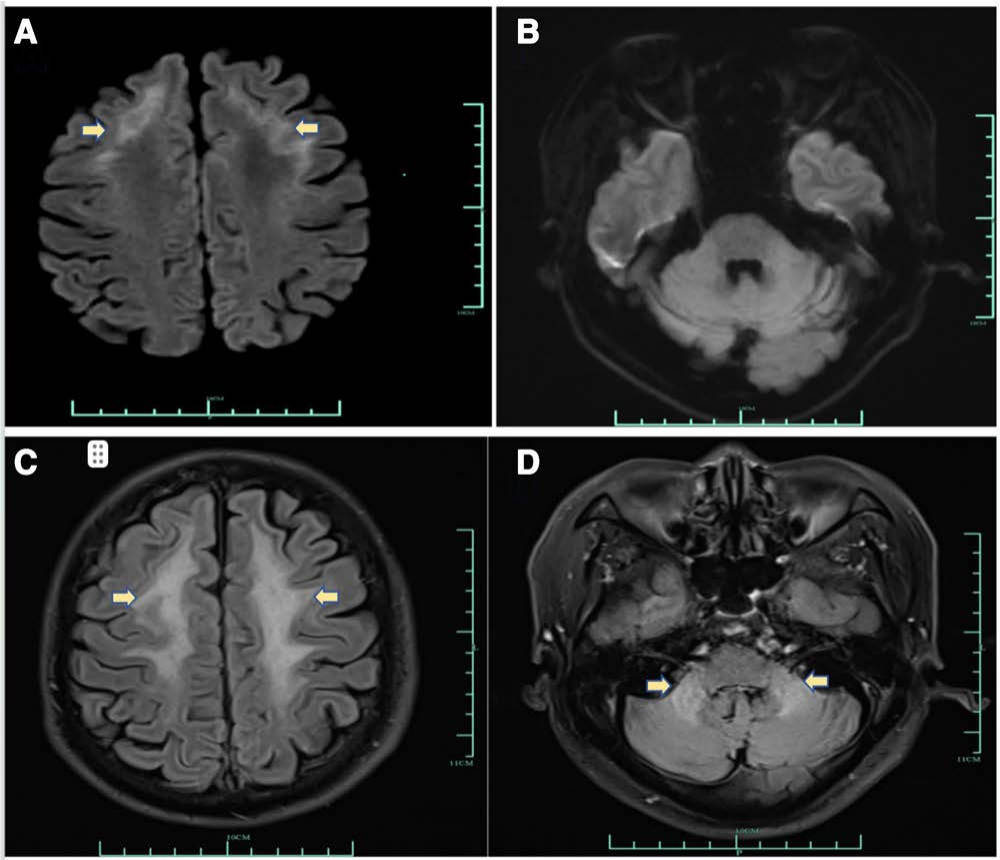
MRI of the brain. (A) MRI diffusion-weighted imaging (DWI) showing hyperintensities in the bilateral frontoparietal corticomedullary junction (yellow arrow).(B) MRI diffusion-weighted imaging (DWI) showing isointensity in the bilateral middle cerebellar peduncles. (C) MRI T2-fluid attenuation inversion recovery (T2-FLAIR) showing hyperintensities in the bilateral frontoparietal cortico-medullary junction (yellow arrow). (D) MRI T2-fluid attenuation inversion recovery (T2-FLAIR) showing hyperintensities in the bilateral middle cerebellar peduncles (yellow arrow).

**Figure 2.**
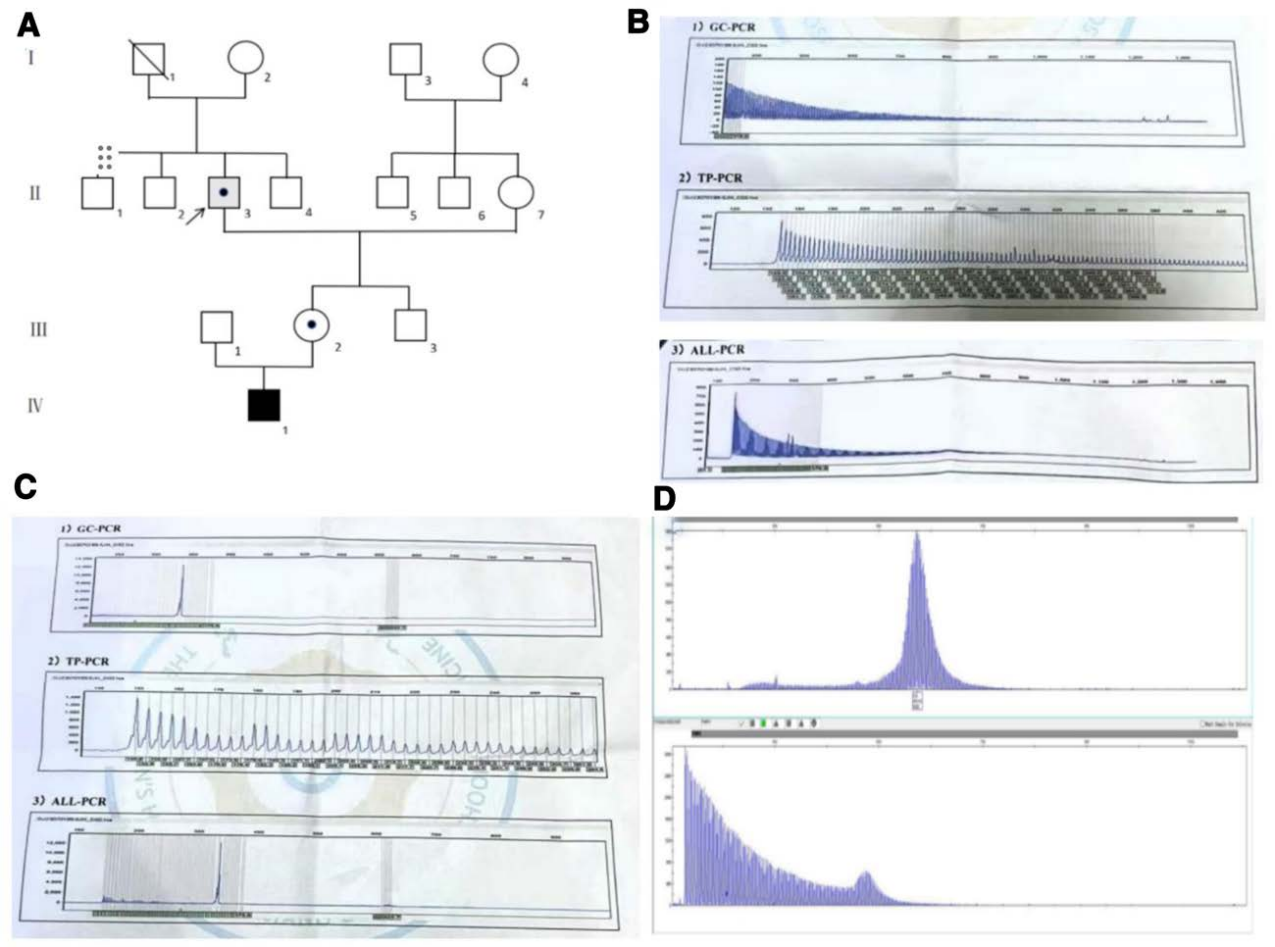
The pedigree and genetic testing results of patient’s family. (A) Pedigree of fragile X-associated tremor ataxia (FXTAS) family. Grey-filled symbols indicate FXTAS patients. Arrow indicates the index case. Black-filled symbols indicate patients with FXS. The dotted symbol indicates FMR1 premutation carriers. Crossed symbols indicate deceased family members (circle: female; square: male). (B) The FMR1 gene result of grandson (Ⅳ-1). (C) FMR1 gene result for the daughter (Ⅲ-2). (D) FMR1 gene result of patient (Ⅱ-3). FXS = fragile X syndrome.

After admission, the brain MRI revealed multiple punctate and patchy signal abnormalities in the bilateral basal ganglia, periventricular regions, and centrum semiovale. These lesions were hypointense on T1-weighted imaging and hyperintense on T2-weighted imaging and T2-fluid-attenuated inversion recovery (T2-FLAIR) sequences. Diffusion-weighted imaging (DWI) also demonstrated linear hyperintense foci in the bilateral frontal subcortical white matter and the splenium of the corpus callosum. The clinical symptoms combined with the characteristic DWI of “flame-shaped” hyperintensities in the bilateral frontoparietal cortico-medullary junction (Fig. 1A) led to clinical suspicion of NIID. However, a skin biopsy revealed neuronal intranuclear inclusions, whereas NOTCH2NLC gene testing was negative. The diagnosis was challenging, and a joint review by the neurology and radiology departments revealed high T2-FLAIR signals in the bilateral middle cerebellar peduncles (MCP sign) (Fig. 1D), suggesting a possible diagnosis of FXTAS. Further family history (Fig. 2A) investigation revealed that the patient’s 7-year-old grandson exhibited symptoms of autism and underwent genetic testing, which showed more than 200 CGG repeats in the FMR1 gene (FM) (Fig. 2B). Further genetic screening showed 120 CGG repeats (premutation carriers) in the daughter (Fig. 2C). Repeat genetic testing revealed 121 CGG repeats (Fig. 2D), confirming the diagnosis of FXTAS. The test revealed a verbal IQ of 74, a performance IQ of 73, and a full scale of IQ 71, and a Clinical Memory Quotient of 74. His Mini-Mental State Examination score was 23, with decrements in short-term memory (only 1 of the 3 elements was recalled correctly). The pedigree of FXTAS family was showed as Figure 2.

The patient was treated with donepezil 2.5 mg/d to improve memory, combined with simvastatin 20 mg/d to regulate lipid levels, and alcohol consumption was restricted. One year later, follow-up revealed that the patient’s memory had improved, allowing him to work as a community security guard, although he occasionally forgot things. A repeated Mini-Mental State Examination was 27, with improved short-term recall of 4 of 5 elements. The patient and his family were actively involved in the treatment and were pleased with the outcomes.

## 3. Discussion

FXTAS is an age-dependent neurodegenerative disorder, with an incidence positively correlated with advancing age, predominantly observed in males.^[6]^ The prevalence of premutation among males over 50 years of age is estimated at approximately 40%.^[7]^

This case involved a 57-year-old male whose symptoms initially included cognitive impairment and occasional lower limb tremor. MRI revealed distinctive symmetrical hyperintensities in the bilateral frontoparietal corticomedullary junction on DWI and MCP sign on high T2-FLAIR. Extensive white matter lesions observed on MRI necessitate considering both NIID and FXTAS. While white matter changes in FXTAS patients are generally less prominent than in NIID, both conditions share similar imaging features. Furthermore, the characteristic MCP sign, typically associated with FXTAS, can also be observed in NIID, making genetic diagnosis crucial for accurate differentiation between the them. Genetic confirmation revealed a CGG repeat expansion of 121, consistent with FXTAS.

FXTAS typically manifests initially as tremor, followed by ataxia and cognitive impairment.^[2,3]^ However, its heterogeneous clinical presentation and variable age of onset pose a diagnostic challenge. In our case, the predominance of cognitive symptoms, coupled with easily overlooked occasional tremor and abnormal hyperintensity at the cortico-medullary junction, initially favored misdiagnosis of NIID. Further in-depth analysis of MCP signs on T2-FLAIR was helpful for differentiation. However, a definitive distinction between FXTAS and NIID relies on genetic testing. Therefore, when middle-aged and elderly male present with cognitive impairment and tremors, especially those with a family history of neurodevelopmental disorders, FXTAS should be included in the differential diagnosis. Literature reports that males with FXTAS over 50 years of age often exhibit cognitive impairment, with early manifestations including deficits in short-term memory and executive function. Some patients, particularly in later stages or with comorbid Alzheimer disease, may progress to clinically overt dementia.^[8]^ This case presented with psychiatric symptoms consistent with subcortical dementia, including psychomotor slowing, poor memory retrieval, mood fluctuations, decreased psychomotor activity, and sleep disturbances. These cognitive deficits may be linked to early psychiatric symptoms such as anxiety and emotional instability. In this case, after treatment with the cholinesterase inhibitor donepezil, both the patient’s cognition and anxiety improved, suggesting that anxiety might be a secondary symptom of the cognitive impairment. Donepezil is FDA-approved for mild to moderate Alzheimer disease and has been shown to improve cognition in neurotypical individuals. While donepezil may not significantly improve cognition in patients with FXS, it has been suggested to normalize FXS-related abnormal neuro-phenotypes.^[9]^ Indeed, Bourgeois et al^[8]^ reported significant cognitive improvement in FXTAS patients treated with donepezil. Furthermore, an animal study demonstrated that intermittent ethanol exposure leads to long-lasting decreases in dendritic spine density and modifies Fmr1 gene expression within the hippocampal formation; importantly, these alterations are ameliorated by donepezil administration.^[10]^ This mechanism may partially explain the observed cognitive improvement in our FXTAS patient with a history of intermittent alcohol use following donepezil treatment.

Targeted invasive genetic testing is required for clinical diagnosis of fragile X-associated diseases. Currently, noninvasive diagnostic methods remain unavailable, making family history particularly critical. In the process of reproduction, the CGG repeats in premutation men do not expand, and their daughters are premutation carriers, which is consistent with the result of the daughter in this case. In females, if one X chromosome carries a fragile X mutation, compensatory expression from the healthy X chromosome may mitigate genetic defects, which may make the symptoms in women milder or even asymptomatic.

FMR1 exhibits dynamic instability, with mutations prone to intergenerational expansion, particularly during maternal transmission. Premutation-carrying females have a 50% chance of transmitting the allele to their offspring, and CGG repeats often expand during the reproductive transmission process,^[2]^ explaining why the patient’s grandson inherited FM. Male premutation carriers show an approximate 47% rate of FXTAS development by their 8th decade. This figure then escalates to 75% once they pass the age of 80.^[7]^ Prior research has predominantly emphasized prenatal counseling on transgenerational inheritance risks to offspring while largely overlooking genetic testing for cross-generational transmission patterns in maternal grandparents.^[2,11]^ When a child is diagnosed with FXS and his mother carries a premutation allele, the maternal grandfather is at risk of developing FXTAS.^[3]^ Early genetic identification empowers proactive risk mitigation by alerting at-risk family members carrying pathogenic alleles to avoid modifiable triggers such as ethanol exposure,^[12]^ potentially delaying or preventing the occurrence of fragile X-associated disorders. However, in clinical practice, most clinicians and families typically limit genetic testing to the affected child and mother, overlooking the fact that the incidence rate in paternal grandfathers is much higher than that in mothers.

Several contributing factors may explain this phenomenon. First, there is a pervasive lack of public awareness regarding the inheritance patterns and disease severity of fragile X-associated disorders. Second, the clinical heterogeneity observed among family members harboring pathogenic FMR1 alleles, ranging from neurodevelopmental impairments to late-onset neurodegeneration, often leads to underreporting due to the oversight of variable phenotypes. The distinct clinical profiles of FXS and FXTAS are mechanistically rooted in divergent pathological mechanisms. FXS is caused by hypermethylation-induced silencing of FMR1, resulting in a deficient FMRP. A lack of FMRP affects synaptic plasticity and neural development, which may lead to intellectual and other cognitive impairments.^[13]^ FXTAS is caused by a premutation allele, which leads to high expression of the FMR1 gene. The pathological mechanism is related to the toxic gain-of-function of FMR1 mRNA.^[14]^ Aberrant FMR1 mRNA leads to the formation of neurotoxic FMRPolyG aggregates. The accumulation of premutation mRNA in neurons triggers the formation of RNA foci and inclusion bodies, leading to neurodegeneration. This can better explain the patient’s cognitive impairment and tremor symptoms, whereas the patient’s grandson mainly presented with autism-like symptoms.^[15]^ The differences in the above-mentioned mechanisms could help clinicians and the public better understand and attach importance to this disease and the significance of multigeneration genetic screening.

## 4. Conclusion

This case serves as a wake-up call. First, when middle-aged and elderly males present with cognitive impairment of unknown cause, clinicians should meticulously document family history and incorporate fragile X-associated testing into diagnostic evaluations. Second, screening only the immediate parental generation for confirmed FXS cases may be insufficient. We also urge DNA testing for the grandparental generation, coupled with targeted educational campaigns to raise awareness. Future research should explore the factors influencing the onset of fragile X-related diseases, providing more evidence-based guidance for premutation carriers to mitigate disease progression. However, given this study’s single-case design and the influence of confounding factors such as hyperlipidemia and alcohol use in the patient, future large-sample cohort studies are warranted. These studies could further explore the etiology and outcomes of unexplained cognitive impairment in middle-aged and older male, and investigate the necessity of multigenerational genetic screening.

## Acknowledgments

The authors thank the patient for consenting to the publication of this report.

## Author contributions

**Data curation:** Yueqi Huang, Wenxin Tang, Xiaoying Zhang.

**Formal analysis:** Yueqi Huang, Ziyang Huang, Zheng Wang, Xiaoying Zhang.

**Writing – original draft:** Yueqi Huang, Xiaoying Zhang.

## References

[1] Salcedo-Arellano MJ Hagerman RJ. Recent research in fragile X-associated tremor/ataxia syndrome. Curr Opin Neurobiol. 2022;72:155–9. 34890957 10.1016/j.conb.2021.11.006

[2] Bourgeois JA. Neuropsychiatry of fragile X-premutation carriers with and without fragile X-associated tremor-ataxia syndrome: implications for neuropsychology. Clin Neuropsychol. 2016;30:913–28. 27355575 10.1080/13854046.2016.1192134

[3] Hall DA Berry-Kravis E. Fragile X syndrome and fragile X-associated tremor ataxia syndrome. Handb Clin Neurol. 2018;147:377–91. 29325626 10.1016/B978-0-444-63233-3.00025-7

[4] Genovese AC Butler MG. Systematic review: fragile X syndrome across the lifespan with a focus on genetics, neurodevelopmental, behavioral and psychiatric associations. Genes (Basel). 2025;16:149. 40004478 10.3390/genes16020149 PMC11855108

[5] Chonchaiya W Nguyen DV Au J. Clinical involvement in daughters of men with fragile X-associated tremor ataxia syndrome. Clin Genet. 2010;78:38–46. 20497189 10.1111/j.1399-0004.2010.01448.x PMC4031089

[6] Grigsby J. The fragile X mental retardation 1 gene (FMR1): historical perspective, phenotypes, mechanism, pathology, and epidemiology. Clin Neuropsychol. 2016;30:815–33. 27356167 10.1080/13854046.2016.1184652 PMC5011753

[7] Jacquemont S Hagerman RJ Leehey MA. Penetrance of the fragile X-associated tremor/ataxia syndrome in a premutation carrier population. JAMA. 2004;291:460–9. 14747503 10.1001/jama.291.4.460

[8] Bourgeois JA Farzin F Brunberg JA. Dementia with mood symptoms in a fragile X premutation carrier with the fragile X-associated tremor/ataxia syndrome: clinical intervention with donepezil and venlafaxine. Neuropsychiatry Clin Neurosci. 2006;18:171–7. 10.1176/jnp.2006.18.2.171 16720793

[9] Bruno JL Hosseini SH Lightbody AA. Brain circuitry, behavior, and cognition: a randomized placebo-controlled trial of donepezil in fragile X syndrome. Psychopharmacol. 2019;33:975–85. 10.1177/0269881119858304 PMC6894490 31264943

[10] Mulholland PJ Teppen TL Miller KM. Donepezil reverses dendritic spine morphology adaptations and fmr1 epigenetic modifications in hippocampus of adult rats after adolescent alcohol exposure. Alcohol Clin Exp Res. 2018;42:706–17. 29336496 10.1111/acer.13599 PMC5903436

[11] Kraan CM Godler DE Amor DJ. Epigenetics of fragile X syndrome and fragile X-related disorders. Dev Med Child Neurol. 2019;61:121–7. 30084485 10.1111/dmcn.13985

[12] Wooden JI Peacoe LE Anasooya Shaji C. Adolescent intermittent ethanol drives modest neuroinflammation but does not escalate drinking in male rats. Cells. 2023;12:2572. 37947650 10.3390/cells12212572 PMC10649200

[13] Richter JD Zhao X. The molecular biology of FMRP: new insights into fragile X syndrome. Nat Rev Neurosci. 2021;22:209–22. 33608673 10.1038/s41583-021-00432-0 PMC8094212

[14] Tseng E Tang HT AlOlaby RR. Altered expression of the FMR1 splicing variants landscape in premutation carriers. Biochim Biophys Acta Gene Regul Mech. 2017;1860:1117–26. 28888471 10.1016/j.bbagrm.2017.08.007 PMC5933929

[15] Shah S Molinaro G Liu B. FMRP control of ribosome translocation promotes chromatin modifications and alternative splicing of neuronal genes linked to autism. Cell Rep. 2020;30:4459–72.e6. 32234480 10.1016/j.celrep.2020.02.076 PMC7179797

